# The effect of pulmonary rehabilitation for post-acute sequelae of SARS-CoV-2 infection in patients: a systematic review and meta-analysis

**DOI:** 10.3389/fresc.2025.1634351

**Published:** 2025-10-31

**Authors:** Yinghua Yue, Xinyi Han, Qiming Chen, Lirong Dai, Qingjuan Ai, Zhigang Zhang, Fangli Ma, Jing Gao

**Affiliations:** ^1^ The First Hospital of Lanzhou University, Lanzhou, China; ^2^ School of Basic Medicine, Gansu University of Chinese Medicine, Lanzhou, China; ^3^ Divsion of Immunology and Respiratory Medicine, Department of Medicine Solna, Karolinska Institutet, Stockholm, Sweden; ^4^ Department of Respiratory Medicine and Allergy, and Center for Molecular Medicine, Karolinska University Hospital, Stockholm, Sweden; ^5^ The First School of Clinical Medicine, Lanzhou University, Lanzhou, China; ^6^ Department of Respiratory Medicine, Gansu Provincial Hospital, Lanzhou, China

**Keywords:** pulmonary rehabilitation, post-acute sequelae of SARS-CoV-2 infection, telerehabilitation, fatigue, exercise capacity

## Abstract

**Background:**

Post-acute sequelae of SARS-CoV-2 infection (PASC), also known as long COVID, are characterized by persistent symptoms such as fatigue, dyspnea, and reduced functional capacity. Pulmonary rehabilitation (PR) is recommended for chronic respiratory conditions, but its effectiveness in PASC, particularly across different delivery modes, remains uncertain.

**Objective:**

To assess the impact of PR, including telerehabilitation and in-person modalities, on physical function, dyspnea, pulmonary function, fatigue, and quality of life in patients with PASC.

**Methods:**

We conducted a systematic search of PubMed, Embase, the Cochrane Library, and Web of Science from inception to March 25 for controlled clinical trials assessing the effects of PR in PASC patients. Two independent reviewers performed study selection and data extraction. The risk of bias was assessed using the Cochrane Risk of Bias Tool, and data were analyzed using Review Manager (RevMan) 5.4.1. Effect sizes were reported as mean differences (MD) or standardized mean differences (SMD) with 95% confidence intervals (CI).

**Results:**

Ten randomized controlled trials involving 673 participants were included. Most studies were judged to have a moderate risk of bias. Compared with usual care, PR significantly improved six-minute walk distance (MD: 76.85 meters; 95% CI: 57.35–96.36; *p* < 0.001), maximal inspiratory pressure (MD: 17.63 cmH₂O; 95% CI: 4.50–30.76; *p* = 0.009), fatigue (SMD: −1.15; 95% CI: −1.83 to −0.48; *p* < 0.001), and quality of life (SMD: 1.73; 95% CI: 0.56–2.91; *p* = 0.004). No statistically significant improvement was found for dyspnea (MD: −0.41; 95% CI: −1.51 to −0.68; *p* = 0.46). Subgroup analyses showed no significant differences between telerehabilitation and in-person PR across all outcomes, including exercise capacity (*p* = 0.84), dyspnea (*p* = 0.86), fatigue (*p* = 0.93), and quality of life (*p* = 0.44).

**Conclusions:**

PR improves physical and functional outcomes in patients with PASC. Telerehabilitation offers a clinically equivalent alternative to in-person PR, supporting its broader implementation.

## 1 Introduction

Coronavirus Disease 2019 (COVID-19) has affected over 510 million individuals and caused more than 6.2 million deaths globally (1). A significant proportion of those infected develop persistent symptoms beyond 12 weeks, known as Post-Acute Sequelae of SARS-CoV-2 Infection (PASC). These symptoms may appear as early as the fourth week post-infection and are not fully explained by alternative diagnoses such as cardiovascular, respiratory, or neurological disorders (2). The estimated global prevalence of PASC is around 43%, suggesting that more than 300 million people may be affected worldwide (3).

PASC is a heterogeneous condition characterized by more than 200 reported symptoms. Patients with symptoms persisting beyond six months frequently report a median of 14 concurrent symptoms (4), with fatigue and dyspnea being the most prevalent and debilitating. These symptoms often co-occur with anxiety, depression, and reduced health-related quality of life (HRQoL), lasting for months or even years (5–7). The complex and overlapping nature of PASC manifestations contributes substantially to the long-term burden on healthcare systems, accounting for up to 30% of COVID-19-related healthcare utilization (3). Without targeted intervention, spontaneous recovery is uncommon, and many patients experience long-term functional impairment and disability (4).

Pulmonary rehabilitation (PR), an evidence-based intervention combining supervised exercise training, education, and psychological support, has been widely recommended to address the multifaceted needs of patients with chronic respiratory diseases. In the context of PASC, PR has demonstrated potential in improving exercise tolerance, pulmonary function, fatigue, mood symptoms, and overall quality of life (8–10). Studies in both chronic obstructive pulmonary disease (COPD) and COVID-19 populations support the efficacy of PR in restoring physical function and reducing psychological burden (11, 12).

Functional limitations are common among PASC patients. One study found that nearly 70% of COVID-19 survivors had six-minute walk distances (6MWD) below predicted values one year after discharge (13), while others reported a 33% reduction in 6MWD compared to healthy controls during 2–6 months of follow-up (14, 15). Moreover, up to 56% of patients with severe initial infection show impaired lung diffusing capacity lasting up to a year. These physical impairments are often accompanied by psychological distress, such as anxiety and depression, in approximately 23% of patients (6, 7), significantly reducing their HRQoL. In response to pandemic-related restrictions and resource constraints, telerehabilitation, the remote delivery of PR via digital platforms, has emerged as a practical alternative to traditional face-to-face programs (16). Preliminary evidence suggests that telerehabilitation may achieve similar outcomes in improving exercise capacity, symptom burden, and quality of life in PASC patients, with additional benefits in accessibility and safety (17, 18). However, the current body of evidence remains limited, heterogeneous, and inconclusive regarding the relative effectiveness of telerehabilitation versus in-person PR.

Therefore, this systematic review and meta-analysis aims to comprehensively evaluate the effectiveness of pulmonary rehabilitation in improving physical and psychological outcomes in patients with PASC and to compare the clinical benefits of telerehabilitation and in-person PR delivery models.

## 2 Methods

The study followed the Preferred Reporting Items for Systematic Reviews and Meta Analysis Protocols checklist (19), the detailed was in Supplementary Table S2. The review was not registered.

### 2.1 Search strategy

A comprehensive and systematic literature search was conducted in four major electronic databases: PubMed, Embase, Web of Science, and the Cochrane Library. The search covered studies published from database inception through March 2025. To ensure both sensitivity and specificity, the search strategy incorporated a combination of controlled vocabulary [Medical Subject Headings (MeSH) for PubMed and Emtree terms for Embase] and free-text keywords. Boolean operators “AND” and “OR” were used to combine terms across three core conceptual domains: the target condition [Post-Acute Sequelae of SARS-CoV-2 Infection (PASC)], the intervention [pulmonary rehabilitation (PR) and its variants], and the study design (randomized controlled trials).

Terms representing the disease condition included “post COVID-19 syndrome,” “long COVID,” “post-acute COVID-19 syndrome,” “chronic COVID syndrome,” “post-acute sequelae of SARS-CoV-2 infection,” “long haul COVID,” “post COVID-19 condition,” “persistent COVID symptoms,” and “PASC.” Terms related to the intervention included “pulmonary rehabilitation,” “respiratory rehabilitation,” “breathing training,” “respiratory muscle training,” “inspiratory muscle training,” “expiratory muscle training,” “breathing exercise,” “airway clearance technique,” “respiratory therapy,” “physical therapy modalities,” and “exercise therapy.” Terms used to define eligible study designs included “randomized controlled trial,” “randomised controlled trial,” “randomized trial,” “clinical trial,” and “RCT.”

No restrictions were placed on language or publication status. The detailed search strategy was in Supplementary Table S1. To supplement the electronic database search, the reference lists of all included studies and relevant systematic reviews were screened manually to identify additional eligible articles not captured by the database queries.

### 2.2 Study selection and eligibility criteria

All retrieved records were imported into EndNote X9 reference management software for deduplication. After the removal of duplicates, two independent reviewers conducted a two-stage screening process. First, titles and abstracts of all identified articles were screened to exclude irrelevant publications. Next, full-text versions of potentially eligible articles were retrieved and reviewed in detail according to pre-defined inclusion and exclusion criteria. Any disagreements between the two reviewers were resolved through discussion, and if consensus could not be reached, a third reviewer was consulted to adjudicate.

Studies were eligible for inclusion if they met the following criteria. The population of interest comprised adult participants (aged 18 years or older) diagnosed with PASC, long COVID, or a similar condition defined as persistent symptoms following laboratory-confirmed or clinically suspected COVID-19 infection. The intervention had to be a structured PR or breathing exercises (exercises to strengthen respiratory muscles, breathing control exercises, airway clearance techniques, or thoracic expansion, etc.), multicomponent exercises (integrated of aerobic, strength, resistance, or endurance exercises, etc.), and Comprehensive PR (both breathing exercises and multicomponent exercises).Both in-person(face-to-face) and telerehabilitation formats were considered eligible. The comparator could be usual care, a sham intervention, or no intervention. Eligible studies had to report at least one quantitative outcome, such as physical function [e.g., six-minute walk distance (6MWD)], dyspnea, pulmonary function parameters [e.g., maximal inspiratory pressure (MIP)], fatigue, or health-related quality of life. Only studies employing an RCT design were included.

Studies were excluded if they were non-randomized designs, included pediatric populations, lacked a comparator group, did not include PR as the primary intervention, or did not provide extractable data for meta-analysis. Conference abstracts, editorials, commentaries, and narrative reviews were also excluded.

### 2.3 Data extraction and risk of bias assessment

Two independent reviewers extracted data using a standardized form. Extracted variables included first author, year of publication, country, study design, sample size, participant demographics, timing of PR initiation, intervention duration and modality, comparator type, outcome measures, and results. Risk of bias was assessed using the Cochrane Risk of Bias Tool version 2.0 (RoB 2), which evaluates bias across domains including random sequence generation, allocation concealment, blinding of participants and outcome assessors, incomplete outcome data, and selective reporting. Each domain was rated as “low,” “some concerns,” or “high.” Discrepancies between reviewers were resolved through discussion or consultation with a third reviewer.

### 2.4 Outcome measures and statistical analysis

The primary outcome was exercise capacity, assessed by the 6MWD. Secondary outcomes included dyspnea measured using the mMRC scale, fatigue assessed through standardized fatigue-related scales, health-related quality of life evaluated via instruments such as EQ-5D or SF-36, and pulmonary function measured by MIP. Meta-analyses were conducted using Review Manager (RevMan) version 5.4. Continuous outcomes were pooled using either mean difference (MD) or standardized mean difference (SMD) with 95% confidence intervals (CI). Heterogeneity was assessed using the I^2^ statistic. Values of I^2^ greater than 50% were considered indicative of substantial heterogeneity, in which case a random-effects model was used. Pre-specified subgroup analyses were performed to compare the effects of telerehabilitation and face-to-face PR. A *p*-value less than 0.05 was considered statistically significant.

## 3 Results

### 3.1 Include studies

**Figure 1:**
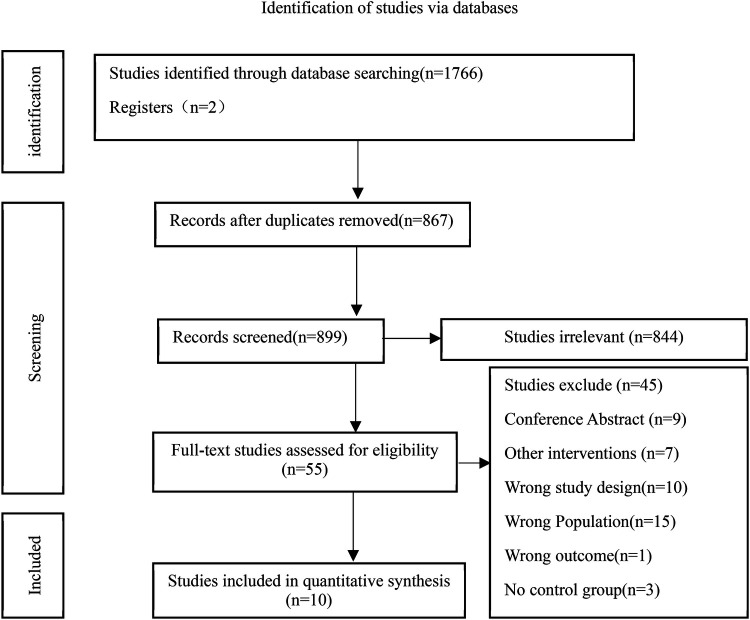
Literature screening flowchart.

The literature search initially retrieved 1,766 records. After removing 899 duplicates, 867 unique records remained. Title and abstract screening led to the exclusion of 844 articles that did not meet the inclusion criteria. A total of 55 full-text articles were assessed for eligibility. Of these, 45 were excluded due to reasons such as non-randomized study design, ineligible population, lack of pulmonary rehabilitation as a primary intervention, or insufficient outcome data. Finally, 10 randomized controlled trials evaluating the effects of pulmonary rehabilitation in individuals with PASC met the inclusion criteria and were included in the systematic review and meta-analysis (Figure 1).

### 3.2 Summary of main results

Tables 1–3 includes descriptive information for the 10 studies included in the systematic review. The studies were performed in China (1), Iran (1), Spain (2), and Egypt (2). UK (1), Turkey (2), and the United States of America (1). All studies were published from 2021 to 2025. Including a total of 673 participants, the ages of the included participants ranged between 23 and 55 years. The participants studied for an average duration of 3 months after COVID-19. The primary symptoms reported by the studies were dyspnea, fatigue, 6MWD, and quality of life. Each study used a different protocol for PR. Face-to-face PR was conducted in a hospital or clinic in 3 RCTs (20, 22, 27) and telerehabilitation PR (e.g., via telerehabilitation tools, videoconference, or phone call) was conducted in 7 RCTs (17, 21, 23–26, 28). Telerehabilitation models were unsupervised home-based (17, 24) or tele-supervised home-based models (21, 23, 25, 26, 28). In total, 5 RCTs (17, 21, 25, 26, 28) included patients previously hospitalized due to COVID-19 infection. One RCT (27) included patients who had not been hospitalized following COVID-19 infection. 4 RCTs (20, 22–24) included a mixed population of both patients. 7 studies (17, 20, 22, 24–26, 28) showed that the included subjects had dyspnea. Among them, six studies (17, 20, 22, 25, 26, 28) used the MMRC scale for grading, and most of the included subjects had a dyspnea degree of grade II∼III. One study (24) adopted the K-BILD questionnaire for assessment, but did not specifically describe the degree of dyspnea in the patients.

**Table 1:** Demographic details of the included studies.

Study	Group	Age	Sex	BMI	Smoker	Former smoker	Days in hospital	Clinical course			Dyspnea	Cough	Fever	Fatigue	Brain fog	Myalgia
			(M/F)		(*n*,%)	(*n*,%)		Mild (n,%)	Moderate (n,%)	Severe (n,%)	(*n*,%)	(*n*,%)	(*n*,%)	(*n*,%)	(*n*,%)	(*n*,%)
del Corral et al. (20)	Intervention	49.0 ± 10.4	12/20	28.08 (6.3)	10 (31)	4 (12)	-	11 (34)	15 (47)	6 (19)	32 (100)			32 (100)	20 (62)	22 (69)
	Control	51.4 ± 10.6	11/21	28.7 (5.2)	11 (34)	6 (19)	-	8 (25)	15 (47)	9 (28)	32 (100)			32 (100)	31 (66)	19 (59)
Deldar et al. (21)	Intervention	54.5 ± 12.0	-	28.1 (5.2)	4 (13.3)		6.6 (2.8)									
	Control	50.4 ± 12.3	-	27.6 (3.8)	1 (3.3)		6.5 (2.9)									
Elyazed et al. (22)	Intervention	49.17 ± 10.75	27/32	30.1 (0.83)							30 (100)					
	Control	52.03 ± 11.10	26/34	29.1 (4.4)							30 (100)					
Alsharidah et al. (23)	Intervention	23.33 ± 2.71	0/24	22.11 (2.66)												
	Control	22.58 ± 2.51	0/24	21.65 (2.73)												
McNarry et al. (24)	Intervention	46.76 ± 12.03	2/35	27.64 (6.80)												
	Control	46.13 ± 12.73	16/95	27.81 (5.83)												
Pehlivan et al. (25)	Intervention	52.77 ± 13.91	14/3	27.98 (21–36)	7 (41)							10 (59)	12 (71)			6 (35)
	Control	44.45 ± 13.36	11/6	27.43 (19–36)	4 (24)							11 (65)	7 (41)			5 (29)
Okan et al. (26)	Intervention	48.85 ± 10.85	15/11	31.14 (4.18)	2 (7.7)	4 (15.4)	9 (0–15)									
	Control	52.19 ± 14.84	12/14	31.31 (4.36)	2 (7.7)	2 (7.7)	9.5 (5–13.75)									
Almazán et al. (27)	Intervention	44.6 ± 9.9.	13/6					19 (100)			11 (57.9)	3 (15.8)	4 (21.1)	16 (84.2)	11 (61.1)	8 (42.1)
	Control	46.0 ± 9.5	16/4					20 (100)			12 (60.0)	3 (15.0)	3 (15.0)	16 (80.0)	10 (50.0)	10 (50.0)
Capin et al. (28)	Intervention	52 ± 10	13/15	34 (9)			5 (3)			7 (25)						
	Control	54 ± 10	5/8	36 (11)			8 (9)			2 (15)						
Li et al. (17)	Intervention	49.17 ± 10.75	27/32		9 (15.3)											
	Control	52.03 ± 11.1	26/34		6 (10.0)											

**Table 2:** PR details of the included studies.

Study	Participants	Intervention type	Timing of PR initiation	Supervision	Dyspnea severity
del Corral et al. (20)	Had been diagnosed with SARS-CoV-2 infection confirmed by polymerase chain reaction (PCR) testing, and presented with long-term post-COVID-19 symptoms, including fatigue and dyspnea	Comprehensive PR (aerobic + resistance + breathing)	>3 months post-infection	Supervised	Dyspnea was inclusion criteria. Severity not numerically specified.
Deldar et al. (21)	With a definite diagnosis of COVID-19 and a positive COVID test showing lung lesions (confirmed by radiographic report or CT scan by an infectious disease specialist), were included.	Comprehensive PR (aerobic + resistance + breathing)	1–3 months post-infection	Remote Supervised	–
Elyazed et al. (22)	Diagnosed and confirmed via COVID-19 polymerase chain reaction (PCR) test within the last three months, either hospitalized or receiving home treatment but not needing ICU admission; discharged at least one month of acute phase recovery with post COVID-19 fatigue, dyspnea, and exercise intolerance	Comprehensive PR (aerobic + resistance + breathing)	1–3 months post-infection	Supervised	Assessment of dyspnea by mMRC
Alsharidah et al. (23)	Diagnosis of COVID-19;included mild to moderate post-COVID-19 survivors, COVID-19 severity was classified into three categories mild to moderate (outpatients with a flu-like condition or probable pneumonia), severe (hospitalized patients treated in hospital wards), and critical (patients treated in an intensive care unit)	Comprehensive PR (aerobic + resistance + breathing)	>3 months post-infection	Supervised	–
McNarry et al. (24)	9.0 ± 4.2 months post-acute COVID-19 self-reported COVID-19 infection	Inspiratory Muscle Training (IMT)	<1 month post-infection	Unsupervised	(K-BILD) questionnaire Severity not numerically specified.
Pehlivan et al. (25)	Diagnosed with COVID-19 and discharged after treatment, still in the first 4 weeks after discharging, described by regression in physical functions after discharge compared to preillness,	Comprehensive PR (aerobic + resistance + breathing)	1–3 months post-infection	Remote Supervised	Assessment of dyspnea by mMRC ranged from 0 to 2
Okan et al. (26)	Received treatment for Covid-19, completed 2months after treatment and presented to the Chest Diseases Outpatient Clinic with dyspnea.	Breathing exercises only	Unspecified	Remote Supervised	Assessment of dyspnea by mMRC ranged from 0 to 4
Almazán et al. (27)	Confirmed microbiological diagnosis of COVID-19 by SARS-CoV2 reverse transcription-polymerase chain reaction on an oropharyngeal–nasopharyngeal swab or a positive rapid antigen test, who presented a chronic symptomatic phase, lasting >12weeks from the onset of symptoms, and had not been hospitalized because of the acute COVID-19 infection.	Comprehensive PR (aerobic + resistance + breathing)	1–3 months post-infection	Supervised	–
Capin et al. (28)	Confirmed SARS CoV-2 infection defined by positive PCR testing, completed a hospitalisation that was at least 24 h	App-based multimodal programs	1–3 months post-infection	Supervised	Not quantitatively reported MRC dyspnea Score
Li et al. (17)	Discharged from one of the participating hospitals after inpatient treatment for COVID-19	Comprehensive PR (aerobic + resistance + breathing)	<1 month post-infection	Unsupervised	mMRC dyspnea score of 2–3. (exact score range not reported)

**Table 3:** PR details of the included studies.

Study characteristics					Intervention				
Study	Year	Sample	Site	Delivery mode	Frequency	Weeks	Experimental group	Control group	Outcomes
del Corral et al. (20)	2025	64 (32/32)	Hospital	In-person	3 times/w	8w	Respiratory muscle training two 20-min sub-sessions,; Aerobic exercise (5-min warm-up, 28-min interval AE training on bicycle ergometer, 8-min cool-down and 10-min stretching exercise)	AE	④⑤
Deldar et al. (21)	2024	60 (30/30)	Home	Telerehabilitation	4 times/w	4w	Telerehabilitation PR (Respiratory rehabilitation exercises 30-minute session per day)	A training booklet an incentive spirometer	①③④⑤
Elyazed et al. (22)	2024	68 (34/34)	Hospital	In-person	5times/w	12w	PR (Respiratory muscle training: 10–15 min, twice per day, daily for 3 months + Resisted training: 3 sets of 10 repetitions, twice a day, 5–7 days a week + Regular walking for 30–60 min, 5 days a week, at a normal pace for 3 months)	No exercise	①③④②
Alsharidah et al. (23)	2023	48 (24/24)	Home	Telerehabilitation	3 times/w	6w	Telerehabilitation PR (breathing exercises and chest expansion, including diaphragmatic breathing exercise, chest mobility for 15 min, Aerobic activity for 20 to 30 min; resistance training for 30 min.)	Written instructions	①
McNarry et al. (24)	2022	148 (111/37)	Home	Telerehabilitation	3 times/w 2 sessions/d	8w	Telerehabilitation (Breathing exercises at 80% of maximal inspiratory pressure with an inspiratory flow device.)	Waitlist.	④⑤
Pehlivan et al. (25)	2022	34 (17/17)	Home	Telerehabilitation	3 times/w	6w	Telerehabilitation PR (patient education, paced running/self-walking on the corridor, breathing exercises, active cycle of breathing technique, range of motion exercise, and standing squat)	Exercise training, similar exercise as the TeleGr by smartphone	②③④
Okan et al. (26)	2022	52 (26/26)	Home	Telerehabilitation	_	5w	Telerehabilitation Breathing Exercises (Respiratory control, pursed lip breathing, and diaphragmatic breathing exercises) + aerobic exercise (mild-intensity walking)	Usual care	①②
Almazán et al. (27)	2022	39 (19/20)	Medical center	In-person	3 times/w	8w	The resistance training, intensity, and intra-set volume were kept constant throughout the training, and a weekly linear volume was varied.	Support for Rehabilitation	②③
Capin et al. (28)	2022	41 (28/13)	Home	Telerehabilitation	_	6w	Telerehabilitation PR (breathing and clearance techniques, high-intensity strength training,16 aerobic and cardiovascular exercise, balance exercises, functional activities, stretching)	Educational handout, safety monitoring, physical activity,	②
Li et al. (17)	2021	119 (59/60)	Home	Telerehabilitation	3∼4 times/w	6w	Telerehabilitation PR: Breathing and thoracic expansion, aerobic exercise and LMS exercises (Aerobic exercise was based on HR reserve determined by Karvonen's formula.)	Short educational instructions at baseline.	①

① six-minute walk test (6-MWT); ② Dyspnea (mMRC,); ③ Fatigue (fatigue related scale); ④ Qualify of life (qualify of life related scale); ⑤ MIP.

The interventions and controls were shown in Table 1. The duration of PR ranged from 4 weeks to 12 weeks, 1 RCT (21) had a duration ⩽4 weeks, 8 RCTs (17, 20, 23–28) had a duration with 4–8 weeks, 1 RCT (22) had a duration of >8 weeks. The timing of PR initiation in 2 RCTs (20, 23) was 3 months after infection,5 RCTs (21, 22, 25, 27, 28) was 1–3 months post-infection,2 RCTs (17, 24) was <1 month post-infection, In addition, 1 RCT (26) was not described. Breathing exercises were performed in 1 RCT (26), Inspiratory Muscle Training were performed in 1 RCT (24), Comprehensive PR (aerobic + resistance + breathing) were performed in 7 RCTs (17, 20–23, 25, 27) and App-based multimodal programs were performed in 1 RCT (28). PR programs varied in the number of sessions and intervention approaches employed. The control group received usual care, no treatment, was given an educational brochure explaining breathing exercises and self-management guidelines, or used a sham device.

### 3.3 Risk of bias in included studies

The quality evaluation results of the RCTs are showed, 6 RCTs (17, 22, 24–26, 28) showed a high risk of bias, 2 (23, 27) showed some concern, and 2 (20, 21) had a low risk of bias. Among the included RCTs, 3 RCTs (20, 21, 23) were performed with double blinding, and the others were performed with single blinding, no blinding, or had an unclear design.

**Figure 2:**
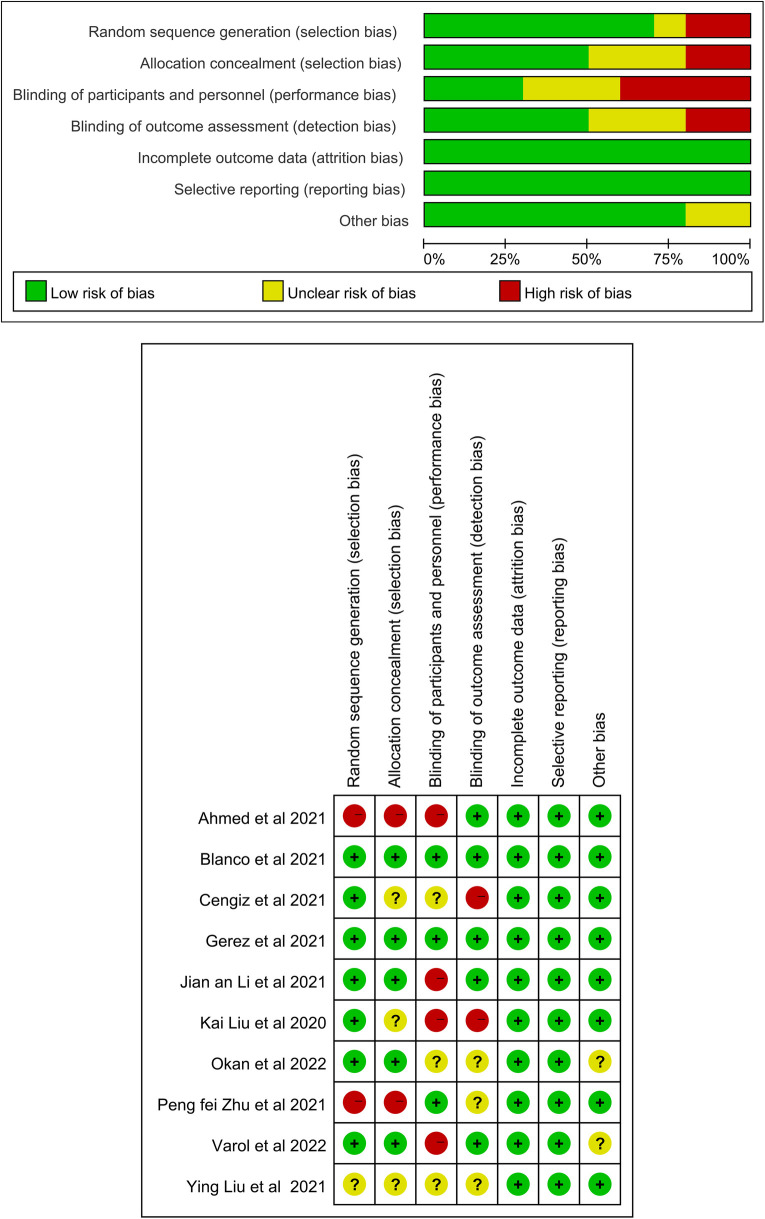
Risk of bias assessment for the randomized trials included in the meta-analysis. **(A)** Risk of bias summary; **(B)** Risk of bias graph. Symbols. (+): low risk of bias; (?): unclear risk of bias; (–): high risk of bias.

The risk of bias across studies was generally moderate. Due to the nature of the intervention, blinding of participants and personnel was not feasible in most trials, potentially introducing performance bias. In addition, blinding of outcome assessment was inconsistently reported, which may have contributed to detection bias. Overall, methodological limitations were noted primarily in the domains of blinding. Blinding participants and therapists is inherently a challenge in rehabilitation research. The detailed risk of bias assessments are shown in Figures 2.

### 3.4 Effects of pulmonary rehabilitation compared with usual care

**Figure 3:**
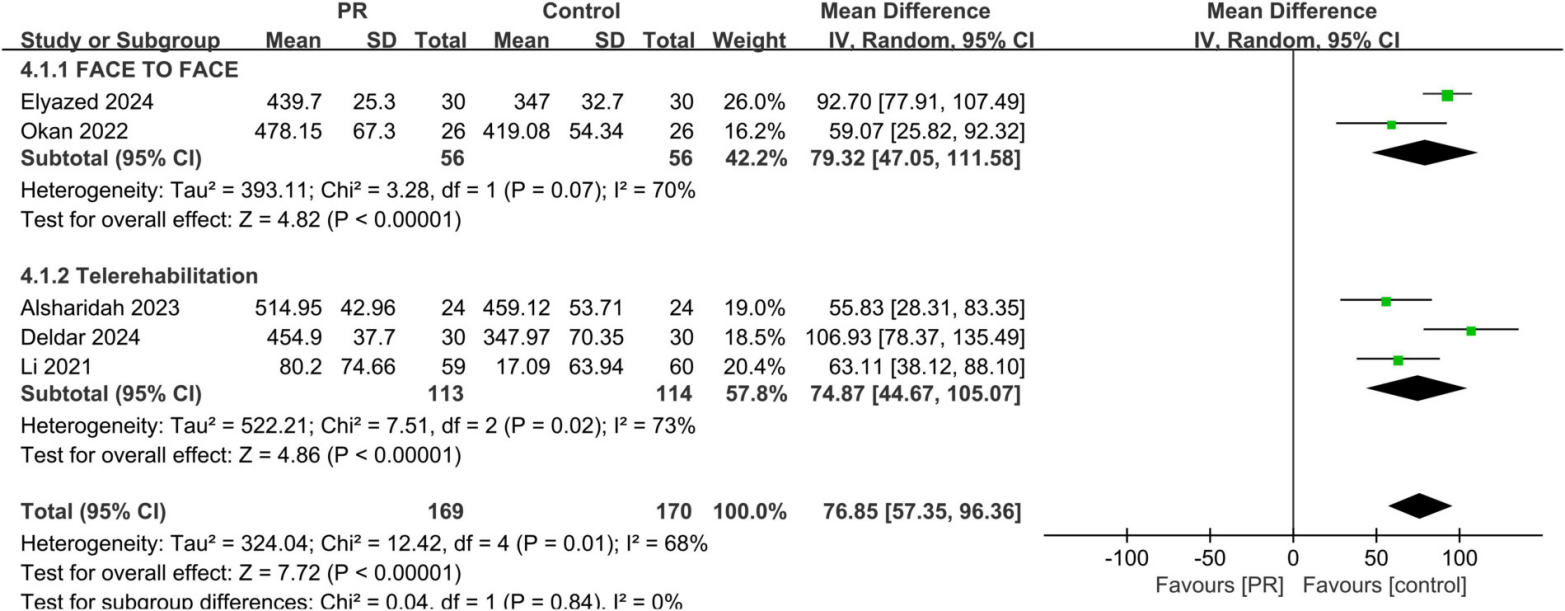
Effect of pulmonary rehabilitation vs. control on exercise capacity (measured by 6-MWT).

Five studies assessed exercise capacity using the 6MWD. Meta-analysis showed that PR significantly improved 6MWD distance compared to usual care, with a mean difference of 76.85 meters (95% CI: 57.35–96.36, *p* < 0.001). Heterogeneity was substantial (*I*^2^ = 68%). The observed effect exceeded the minimal important difference of 30 meters, suggesting both statistical and clinical relevance (29, 30) shown in Figure 3.

**Figure 4:**
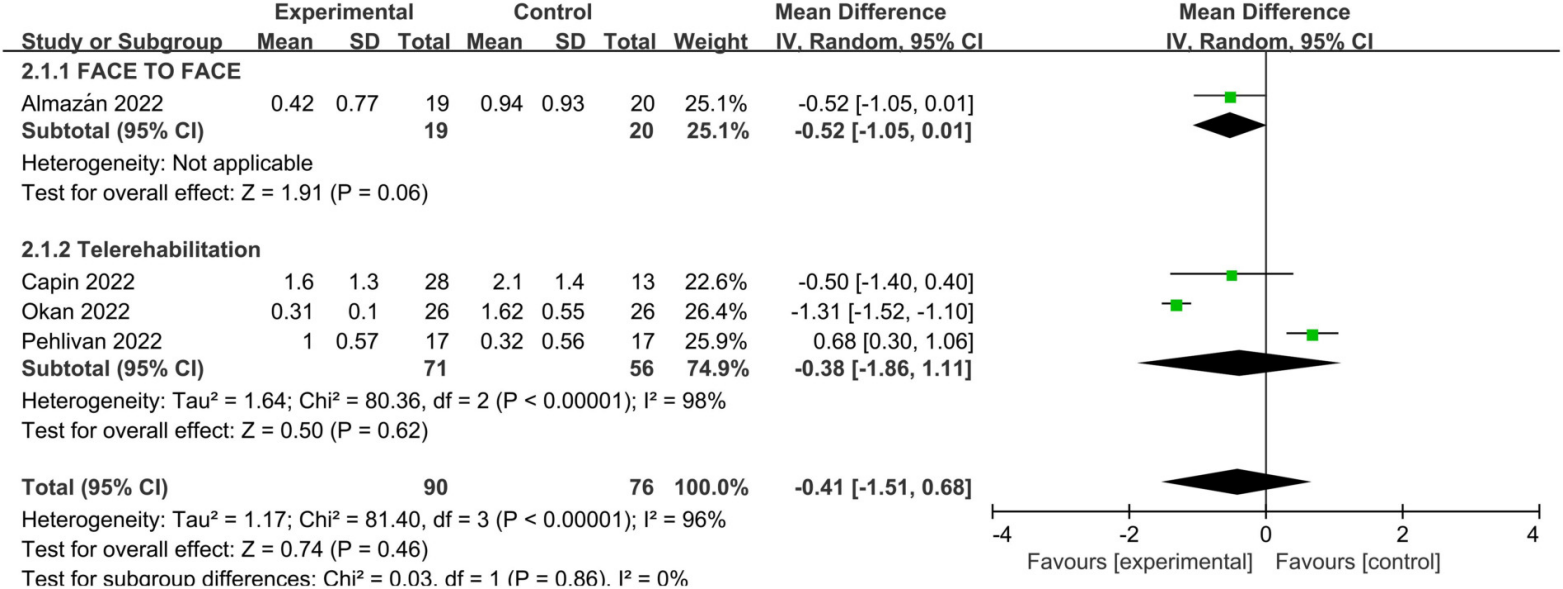
Effect of pulmonary rehabilitation vs. control on dyspnea (measured by mMRC).

Dyspnea was evaluated using the mMRC scale in four studies. The pooled mean difference favored PR but was not statistically significant (MD: −0.41, 95% CI: −1.51 to 0.68, *p* = 0.46). Heterogeneity was considerable (*I*^2^ = 96%). One additional study reported a statistically significant within-group improvement in dyspnea (*p* < 0.008) but could not be included in the meta-analysis due to untransformable data (22) shown in Figure 4.

**Figure 5:**
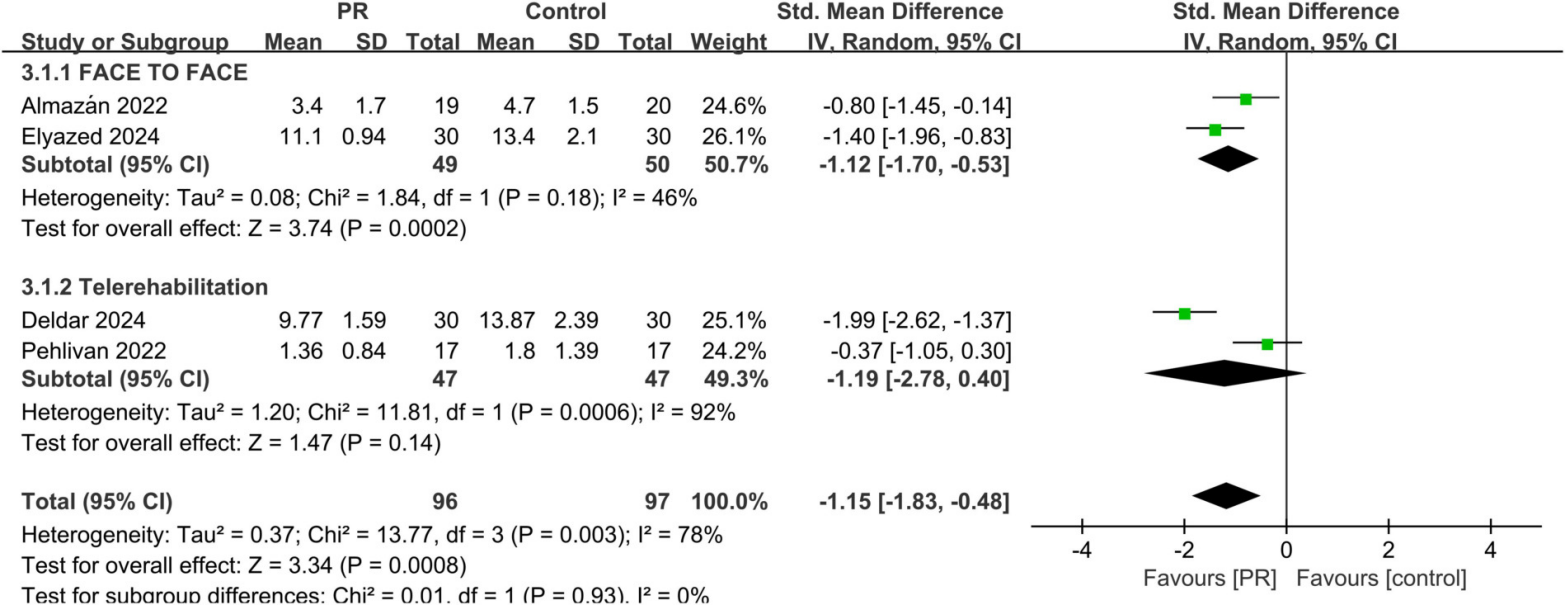
Effect of pulmonary rehabilitation vs. control on fatigue.

Fatigue was assessed in four studies using validated scales. Meta-analysis showed a significant reduction in fatigue among patients receiving PR (SMD: −1.15, 95% CI: −1.83 to −0.48, *p* < 0.001), with high heterogeneity (*I*^2^ = 78%) shown in Figure 5.

**Figure 6:**
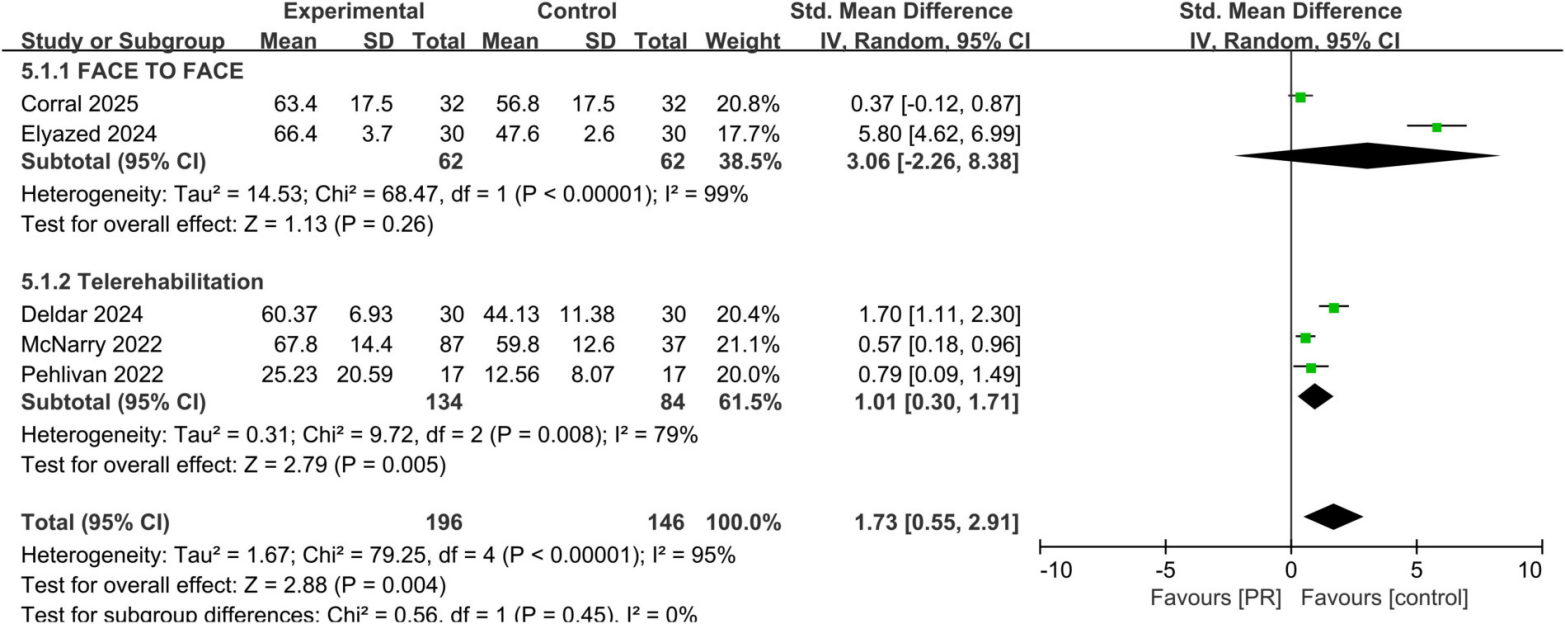
Effect of pulmonary rehabilitation vs. control on the quality of life.

Five studies assessed health-related quality of life using instruments such as the EQ-5D and SF-36. Pooled analysis demonstrated a significant improvement following PR (SMD: 1.73, 95% CI: 0.55–2.91, *p* = 0.004), though heterogeneity remained high (*I*^2^ = 95%) shown in Figure 6.

**Figure 7:**
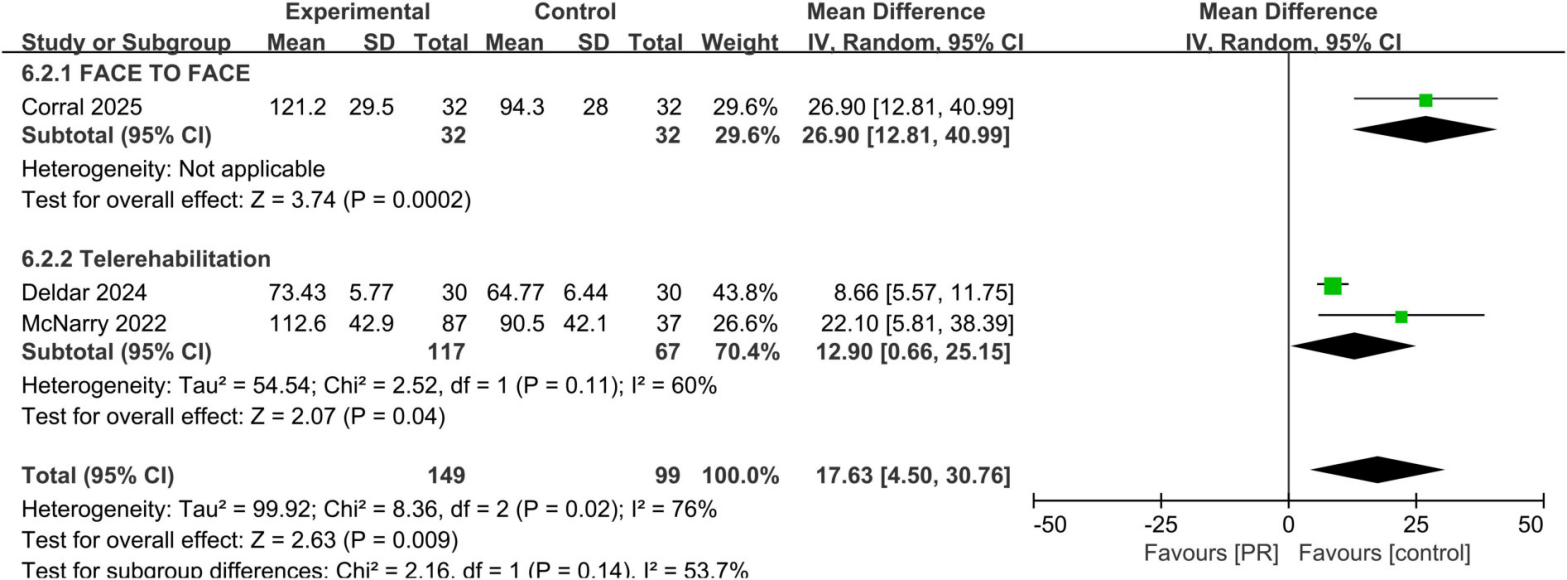
Effect of pulmonary rehabilitation vs. control on MIP.

Pulmonary function was evaluated in three studies using MIP. The pooled results showed a significant improvement in MIP in the PR group compared to controls (MD: 17.63 cmH_2_O, 95% CI: 4.50–30.76, *p* = 0.009), with moderate-to-substantial heterogeneity (*I*^2^ = 76%) showed in Figure 7.

### 3.5 Comparison between telerehabilitation and face-to-face pulmonary rehabilitation

Subgroup analyses comparing telerehabilitation and face-to-face PR revealed no statistically significant differences in clinical outcomes. For exercise capacity, the between-group *p*-value was 0.84; for dyspnea, *p* = 0.86; for fatigue, *p* = 0.93; and for health-related quality of life, *p* = 0.44. In the analysis of pulmonary function, no heterogeneity was observed among face-to-face PR studies (*I*^2^ = 0%), whereas telerehabilitation studies exhibited substantial heterogeneity (*I*^2^ = 76%) shown in Figure 7.

## 4 Discussion

The ten studies included in this review utilized respiratory muscle training or combined endurance-based PR protocols. Comprehensive meta-analytic data demonstrated that PR significantly improved exercise capacity, as measured by the 6MWD, and enhanced pulmonary function, as indicated by MIP. Despite heterogeneity in the assessment tools used across studies for dyspnea, fatigue, and HRQoL, pooled analyses suggested that PR had favorable effects on all three domains. These findings align with prior studies on PR in patients with chronic respiratory diseases and in those recovering from acute COVID-19 infection (12, 31).

Jenkins et al. reported that PR was associated with a reduced risk of hospital readmission in patients with COPD (odds ratio 0.48, 95% CI: 0.30–0.77), as well as improved 6MWD (mean difference 57 m), HRQoL, fatigue, and dyspnea scores (31). Additional studies have confirmed that PR reduces post-discharge morbidity and mortality in COPD and other chronic respiratory diseases (32). The benefits of PR have also been documented in interstitial lung disease, pulmonary nodular disease, and pulmonary hypertension (33, 34).

PR improves overall cardiopulmonary fitness by enhancing the functional performance of respiratory and peripheral muscles, increasing respiratory compliance, reducing the work of breathing, and facilitating the clearance of inflammatory or fibrotic pulmonary lesions (35–38). Notably, the present review found a pooled improvement in 6MWD of 76.85 meters in patients with PASC, which is substantially greater than the average 40-meter improvement typically reported in patients with chronic lung disease (11, 39). These findings not only meet but exceed the minimal important difference threshold of 30 m in 6MWD seen in patients with COPD or interstitial lung disease, underscoring the clinical relevance of PR for patients with PASC.

With respect to fatigue, our findings indicate that PR is effective in reducing fatigue symptoms. However, emerging literature suggests that patients with severe fatigue may benefit most from individualized, multidisciplinary rehabilitation programs combining cardiorespiratory training with psychosocial support (40, 41). Clinicians should therefore approach PR in PASC with careful clinical characterization, standardized symptom assessments, and biopsychosocial profiling to optimize therapeutic benefit and minimize risks.

Although our analysis found a non-significant reduction in dyspnea as measured by the mMRC scale, this is consistent with findings from Oliveira et al. who also reported no significant improvement (mean difference −0.57; 95% CI: −1.32 to 0.17) (42). Several methodological and clinical factors may account for this observation. First, a floor effect may have influenced the detectability of change. In multiple included trials, participants presented with relatively low baseline levels of dyspnea, often reflected by mMRC scores of ≤1, which limits the measurable scope for further improvement. This effect is particularly relevant when sample sizes are modest or variability is high (43). Second, heterogeneity in dyspnea assessment tools represents a significant methodological limitation. Across studies, dyspnea was evaluated using different instruments, including the mMRC, Borg CR10, and visual analogue scales. These tools differ in their psychometric properties, sensitivity to change, and the dimension of breathlessness they capture (e.g., exertional vs. perceived dyspnea), thus reducing outcome comparability and potentially attenuating pooled effect sizes (Martins et al., 2024). In addition, the timing of rehabilitation initiation and the complex etiology of post-COVID dyspnea, which may involve pulmonary, cardiovascular, neuromuscular, and psychological components, further complicate both measurement and intervention response. Hence, the absence of statistically significant improvement in dyspnea likely reflects a confluence of factors, including baseline symptom intensity, tool heterogeneity, and underlying pathophysiology. Future trials should stratify patients by initial dyspnea severity, employ standardized and multidimensional dyspnea assessment instruments, and consider adjunctive therapies such as breathing retraining, inspiratory muscle training, or cognitive-behavioral interventions to more directly address dyspnea mechanisms in PASC populations.

As PR evolves, telerehabilitation has emerged as an increasingly relevant model. It involves digital delivery of supervise exercise, monitoring, and patient engagement through online platforms. It offers logistical advantages, particularly for individuals with limited access to transportation or care facilities (44). Studies have demonstrated that telerehabilitation can incorporate pre- and post-intervention assessments conducted in clinical settings while enabling real-time exercise sessions through videoconferencing (45, 46). Furthermore, telerehabilitation has the potential to reduce costs, increase patient autonomy, and serve as a maintenance strategy for patients with chronic respiratory diseases (47, 48). Diverse models have been applied, including wearable device–integrated platforms and asynchronous video-based monitoring. Despite this variability, high adherence rates and positive user satisfaction have been consistently reported, supporting telerehabilitation as a practical alternative to traditional center-based PR.

To further contextualize these findings, it is important to consider the unique challenges and opportunities associated with implementing telerehabilitation, particularly in rural or resource-constrained settings. In such environments, conventional PR programs are often inaccessible due to infrastructure limitations, workforce shortages, and geographic barriers. The transition to remotely delivered, home-based PR offers a scalable and adaptable solution that may broaden access, particularly during public health crises such as the COVID-19 pandemic (17, 28, 43). However, its successful deployment relies heavily on stable internet infrastructure, patient digital literacy, and supportive policy environments, factors that may be insufficient in low- and middle-income regions. Moreover, our review highlights substantial heterogeneity across studies in terms of rehabilitation modalities (e.g., aerobic, resistance, or inspiratory muscle training), intervention duration, intensity, supervision, and outcome measures. This variability hinders data synthesis and limits the generalizability of findings. The development of standardized, evidence-based PR protocols tailored to PASC is urgently needed. Such protocols should include core components, delivery frameworks, and validated outcome measures to facilitate both clinical implementation and comparative research (49, 50). Finally, while the short-term benefits of PR, including improvements in exercise capacity, fatigue, and quality of life, have been consistently demonstrated, evidence on its long-term effectiveness remains limited. Most included studies had follow-up durations ranging from 6 to 12 weeks, with few assessing outcomes beyond this timeframe. Longitudinal studies with extended follow-up (≥6 months) are critically needed to evaluate the durability of PR benefits, monitor potential relapse in functional status, and assess cost-effectiveness in diverse healthcare systems (51, 52).

In addition, while PR demonstrates clinical benefit for many individuals recovering from PASC, it may not be universally applicable. Post-COVID-19 condition represents a heterogeneous syndrome, encompassing a wide spectrum of clinical phenotypes involving respiratory, cardiovascular, neurological, musculoskeletal, and psychological domains (53, 54). A uniform rehabilitation approach risks overlooking subgroups whose predominant manifestations—such as cognitive impairment (“brain fog”), autonomic dysfunction, or post-exertional symptom exacerbation (PESE), may not only fail to benefit from traditional aerobic-based PR but may even deteriorate (4, 55). For these individuals, rehabilitation strategies incorporating symptom pacing, neurocognitive support, and energy-conserving physical activity plans may be more appropriate. Despite these distinctions, most current PR trials do not stratify interventions based on patient phenotype or baseline functional status. Moreover, a lack of consensus on standardized rehabilitation protocols and core outcome sets reduces reproducibility and limits clinical generalizability (56). Long-term evidence is also sparse; only a few studies have assessed sustained outcomes beyond 12 weeks, with limited data on relapse, work reintegration, mental health, or healthcare utilization (51). To optimize post-COVID rehabilitation, future research should focus on identifying the subpopulations most likely to benefit from PR, developing flexible yet standardized intervention frameworks, and evaluating long-term efficacy, adherence, and implementation feasibility within real-world healthcare systems.

There is some variability in the telerehabilitation models used across studies, with some researchers incorporating wearable devices synchronized to online platforms, while others used video-based systems to monitor and record patient performance asynchronously. Despite these differences, patient adherence rates were generally high, and most studies reported favorable feedback regarding accessibility and satisfaction with the telerehabilitation format. This supports telerehabilitation as a feasible alternative to in-person PR, though future research is needed to establish optimal delivery protocols and long-term outcomes.

This study performed subgroup analyses stratified by in-person and remote rehabilitation modalities; however, the results continued to exhibit high heterogeneity. The underlying causes may be linked to variations in patient characteristics, assessment tools, intervention timing, and pulmonary rehabilitation (PR) protocols. First, while the baseline characteristics of the included patients did not show statistically significant differences, subgroup analyses based on symptom profiles revealed disparities in responsiveness to PR. Research has demonstrated that PR models focused on respiratory rehabilitation yield superior efficacy in subgroups primarily presenting with dyspnea and cardiopulmonary sequelae (57). In contrast, greater heterogeneity and inconsistent effectiveness were observed in subgroups dominated by primary fatigue and neurocognitive sequelae. This phenomenon may be attributed to the lack of targeted functional exercise interventions for these specific symptoms in existing PR protocols (57, 58). Second, intervention timing constitutes another source of heterogeneity, as the duration of illness and stage of recovery influence PR outcomes. PR interventions produce more consistent effects during the early phase of post-COVID syndrome, where sequelae are often associated with acute-phase physical deconditioning. In the late phase, however, outcomes become more variable. This variability may stem from the complexity of the underlying mechanisms in late-stage sequelae, such as persistent immune dysregulation and autonomic dysfunction (42). Additionally, comorbidities among the included patients may contribute to heterogeneity. Patients with pre-existing respiratory or cardiovascular conditions derive more consistent benefits from PR (59), whereas those with metabolic comorbidities exhibit greater variability in outcomes. This discrepancy could arise because comorbidities affect the body's tolerance of and adherence to PR interventions. Furthermore, differences in intervention protocols contribute to heterogeneity. Variations in intervention combinations, exercise intensity, and frequency all lead to divergent results. In the context of remote PR, disparities in technical support—such as real-time physiological monitoring and dynamic protocol adjustments using smart wearable devices in some studies vs. fixed exercise guidance via video alone in others—directly impact intervention effectiveness due to differing levels of technological empowerment. Moreover, variations in assessment tools represent a source of heterogeneity. Fundamental differences exist in tool focus, scoring methodologies, and measurement dimensions, which may introduce heterogeneity when aggregating results.

All included studies in this research are randomized controlled trials (RCTs), each employing randomization and allocation concealment procedures. However, unmeasured confounding factors persist in group assignment. For instance, clinicians may be more inclined to allocate patients with better baseline exercise capacity or stronger social support to the PR intervention group. Such biases could influence unmeasured variables affecting outcomes, such as treatment adherence. When allocation bias is present, statistical adjustments often fail to account for imbalances in key variables, including symptom severity, comorbidity burden, or prior rehabilitation experience. These residual confounding factors obscure the true effectiveness of PR, leading to either overestimation or underestimation of its efficacy in specific post-COVID syndrome subgroups. Notably, allocation bias and heterogeneity are interconnected: biased allocation may exaggerate outcome differences between subgroups and mask the genuine effects of PR.

In conclusion, clarifying the boundaries of heterogeneity shaped by symptom subgroups, timing of intervention, and comorbidities helps us understand when and for which patients pulmonary rehabilitation models are most effective in post-COVID syndrome. Addressing allocation bias through rigorous randomization, transparent reporting of allocation methods, and robust adjustment for confounding factors is crucial for enhancing the external validity of pulmonary rehabilitation research across diverse populations.

This review has several limitations. First, there was substantial heterogeneity in intervention protocols, duration, and outcome measurement tools across studies, which limits the precision of effect estimates. Second, the overall number of eligible RCTs remains limited, and many included studies had small sample sizes. Third, due to variability in terminology and symptom classification, some potentially relevant studies may have been missed despite comprehensive search efforts. Finally, the included trials lacked long-term follow-up data, making it difficult to assess the sustainability of PR benefits in patients with PASC.

## 5 Conclusion

PR significantly improves exercise capacity, pulmonary function, fatigue, and quality of life in patients with PASC. Although the improvement in dyspnea was not statistically significant, the direction of effect remained favorable. Telerehabilitation appears to be a promising alternative to face-to-face PR, offering comparable clinical benefits while improving accessibility. Future high-quality RCTs with standardized protocols and long-term follow-up are warranted to optimize PR strategies and delivery models in this growing patient population.

## Data Availability

The original contributions presented in the study are included in the article/Supplementary Material, further inquiries can be directed to the corresponding authors.
