# Supplementary material for: The effect of pulmonary rehabilitation for post-acute sequelae of SARS-CoV-2 infection in patients: a systematic review and meta-analysis
Source: Front Rehabil Sci. 2025 Oct 31;6:1634351. doi: 10.3389/fresc.2025.1634351 (PMC12615381; doi:10.3389/fresc.2025.1634351)
Supplement: Supplementary file 1 [file Table1.docx]

Table S1 Search strategy

PubMed

#1 "Breathing Exercises"[Mesh]

"Respiratory Therapy"[Mesh]

#2 (((((("Breathing training"[Title/Abstract]) OR ("Respiratory muscle training"[Title/Abstract])) OR ("respiratory rehabilitation"[Title/Abstract])) OR ("pulmonary rehabilitation"[Title/Abstract])) OR ("Breathing Exercise"[Title/Abstract])) OR ("Inspiratory muscle training"[Title/Abstract])) OR ("expiratory muscle training"[Title/Abstract])OR ("Airway clearance technique*"[Title/Abstract])) OR (Exercise therapy[Title/Abstract])) OR (Physical therapy modalities[Title/Abstract]))

#3 #1 OR #2

"Fatigue Syndrome, Chronic"[Mesh]

"Post-Acute COVID-19 Syndrome"[Mesh]

#4 (long COVID[Title/Abstract]) OR (Post-acute COVID-19 syndrome [Title/Abstract]) OR (Post-COVID condition[Title/Abstract]) OR (Chronic COVID syndrome[Title/Abstract]) OR (Post-acute sequelae of SARS-CoV-2 infection[Title/Abstract]) OR Post-COVID-19 syndrome[Title/Abstract]) OR (Long haul COVID[Title/Abstract]) OR (Post-viral fatigue syndrome AND COVID-19[Title/Abstract])

#5 #3 AND #4
